# Molecular Landscape of Vulvar Squamous Cell Carcinoma

**DOI:** 10.3390/ijms22137069

**Published:** 2021-06-30

**Authors:** Núria Carreras-Dieguez, José Guerrero, Maria Teresa Rodrigo-Calvo, Inmaculada Ribera-Cortada, Isabel Trias, Pedro Jares, Ricardo López del Campo, Adela Saco, Meritxell Munmany, Lorena Marimon, Melania Ferrando, Naiara Vega, Marta del Pino, Aureli Torné, Jaume Ordi, Natalia Rakislova

**Affiliations:** 1 Clinical Institute of Gynecology, Obstetrics, and Neonatology, Hospital Clínic de Barcelona, Universitat de Barcelona, 08036 Barcelona, Spain; ncarreras@clinic.cat (N.C.-D.); mmunmany@clinic.cat (M.M.); mdelpino@clinic.cat (M.d.P.); atorne@clinic.cat (A.T.); 2 Institut d’Investigacions Biomèdiques August Pi i Sunyer (IDIBAPS), Hospital Clínic de Barcelona, 08036 Barcelona, Spain; pjares@clinic.cat; 3 Department of Pathology, Hospital Clínic de Barcelona, Universitat de Barcelona, 08036 Barcelona, Spain; JAGUERRERO@clinic.cat (J.G.); MTRODRIGO@clinic.cat (M.T.R.-C.); itribera@clinic.cat (I.R.-C.); itrias@clinic.cat (I.T.); rilopez@clinic.cat (R.L.d.C.); masaco@clinic.cat (A.S.); nvega@clinic.cat (N.V.); jordi@clinic.cat (J.O.); 4 ISGlobal, Hospital Clínic de Barcelona, Universitat de Barcelona, 08036 Barcelona, Spain; lorena.marimon@isglobal.org (L.M.); melania.ferrando@isglobal.org (M.F.)

**Keywords:** vulvar cancer, vulvar squamous cell carcinoma, molecular analysis, genomic landscape, next generation sequencing, whole-exome sequencing

## Abstract

Vulvar squamous cell carcinoma (VSCC) is a rare malignancy with dual pathogenesis, Human papillomavirus (HPV)-associated and HPV-independent, with a poorly explored molecular landscape. We aimed to summarize the findings of the series analyzing molecular hallmarks of this neoplasm. In January 2021, we conducted a comprehensive literature search using Pubmed Medline and Scopus to identify publications focused on genomic profiling of VSCC. Observational studies, including both prospective and retrospective designs, evaluating molecular alterations in VSCC were deemed eligible. A total of 14 studies analyzing 749 VSCC were identified. The study series were heterogeneous in HPV testing and sequencing strategies, included small sets of tumors and cancer genes, and commonly lacked survival analysis. Only one extensive targeted next-generation sequencing-based study comprised a large cohort of 280 VSCC. The mutated genes, their number, and frequencies were highly variable between the series. Overall, *TP53* and *CDKN2A*, followed by *PIK3CA*, *HRAS*, and *PTEN*, were the most frequently studied and mutated genes. Mutations involved in the PI3K/AKT/mTOR pathway, including *TP53*, *HRAS*, *KRAS*, and *PIK3CA*, have been consistently reported across the studies. However, the role of individual mutations or pathways in the development of VSCC remains unclear. In conclusion, heterogeneity and the small sample size of available molecular series contribute to a limited view of the molecular landscape of VSCC. Large-scale genome- or exome-wide studies with robust HPV testing are necessary to improve the molecular characterization of VSCC.

## 1. Introduction

Vulvar squamous cell carcinoma (VSCC) is an uncommon malignancy of the lower genital tract generally regarded as a disease in older women [1]. However, some epidemiological indicators suggest a rising incidence of this tumor in young women, which added to the increasing life expectancy, will likely cause an increase in the rates of this disease in the future [2]. In the last decade of the 20th century, it became clear that there are two different etiopathogenic pathways leading to VSCC: one associated with human papillomavirus (HPV) and a second carcinogenic pathway independent of HPV infection [3]. A number of studies have provided evidence showing that HPV-associated and HPV-independent VSCC have different clinico-pathological features and natural history [4]. These etiopathogenic and clinical differences between HPV-dependent and HPV-independent tumors have also been seen in other types of tumors that have been studied more than VSCC, such as head and neck carcinomas [5,6]. The geographical distribution of these two types of VSCC is also different [7]: in high-income countries, most VSCC developed through the HPV-independent route [4] and affected mostly post-menopausal women [8], whereas in low- and middle-income countries HPV-associated VSCC were more common [7] and involved younger patients [8].

Classically, VSCCs had been classified according to their morphological features. All previous VSCC classifications included several histological types, namely basaloid, warty, keratinizing and non-keratinizing subtypes, as well as other infrequent variants. The main drawback of these morphology-based classifications was the complete lack of prognostic implications [9]. The publication of the new classification from the World Health Organization (WHO) in September 2020 [10] has resulted in a major conceptual shift in the categorization of VSCC (and also of vaginal and cervical tumors), as for the first time it gives priority to a molecular attribute—i.e., the HPV status—rather than to the histological features. In this new WHO classification VSCC are divided into two major types, HPV-associated and HPV-independent [10]. Increasing evidence indicating that HPV-associated VSCCs have a better prognosis than HPV-independent tumors [5,6] was the rationale leading to this major change in the classification. Nevertheless, despite the clear etiological and clinical differences between these two major types of VSCC, the management of patients with HPV-associated and HPV-independent VSCC remains the same.

There is strong evidence indicating that p16 immunohistochemistry (IHC) can be used as a surrogate marker to establish HPV status in VSCC [11]. Although not perfect, p16 IHC seems to be more reliable than HPV testing, a method that has shown some limitations [9,11,12,13]. The carcinogenic pathways of HPV-associated VSCC are similar to the carcinogenesis of cervical carcinoma, the model for HPV-associated tumors [14]. Most of these HPV-associated tumors arise in an intraepithelial precursor histologically similar to the cervical precursor and is called a high-grade squamous intraepithelial lesion [15]. However, its mutational landscape is not completely understood. Alternatively, the molecular mechanisms leading to HPV-independent VSCC remain unclear and complex. Inflammatory dermatoses, including lichen sclerosus and lichen simplex chronicus, are considered the main etiologic drivers [16]. An intraepithelial precursor, called a differentiated vulvar intraepithelial neoplasia (dVIN), is frequently identified in the adjacent skin and is thought to precede most HPV-independent VSCC [17]. Mutations in *TP53* have been identified in a significant proportion of these tumors [3]. Recently, a different subset of HPV-independent precursors has been described, namely, differentiated exophytic vulvar intraepithelial lesions (DEVIL) [18], and vulvar acanthosis with altered differentiation (VAAD) [19], which seem to be associated with a particular subset of p53 wild type HPV-independent VSCC; these tumors are frequently classified morphologically as verrucous carcinomas. 

Recent advances in next-generation sequencing (NGS) are giving rise to an unprecedented characterization of cancer genomes [20,21]. NGS studies are commonly focused on somatic mutations and copy number variations, major players in cancer development. Molecular research in cancer remains challenging and progress is far more evident in prevalent malignancies, such as breast or lung cancers, than in rare malignancies such as vulvar or penile cancer. 

The mutational landscape of VSCC has been poorly investigated over the past three decades. The vast majority of the research on VSCC has focused mainly on the mutations of the tumor suppressor gene *TP53* [22,23,24] and those genes known to be relevant in head and neck cancer [25]. In contrast, large-scale whole-genome- or whole-exome sequencing studies in VSCC have been absent in the past few decades. Thus, knowledge on the molecular hallmarks of HPV-associated and HPV-independent VSCC is limited to date. 

Knowledge on recurrent mutations in VSCC will certainly open doors to better prognostic stratification and the identification of new targets for therapy. Herein, we aimed to review the existing molecular-based study series on VSCC, provide an overview of the available genomic data, and present challenges in the molecular characterization of VSCC.

## 2. Methodology

In January 2021, we conducted a comprehensive literature search using Pubmed Medline and Scopus to identify publications focused on genomic profiling of VSCC. We used the terms “vulva”, “cancer”, “carcinoma”, “molecular”, “genomic”, and “mutation”. Observational studies, including both prospective and retrospective designs, evaluating molecular alterations in VSCC were deemed eligible. Reviews, meta-analyses, and letters to editors, as well as publications in languages other than English, were excluded. Reference lists from initially selected studies and from reviews were searched to identify additional relevant studies. Selected articles were additionally cross-referenced. Studies in which data on the frequency of the mutated genes were not specifically reported were excluded. Additional exclusion criteria involved articles focusing on non-squamous cell neoplasms, those analyzing only chromosome arm-level alterations, and those not specifically focused on the VSCC molecular landscape. 

Study selection was based on the content of the abstract. Two reviewers (NR and NC) independently evaluated the papers. Studies focused on genomic alterations in VSCC were selected. The full text of the articles was, then, reviewed to ensure they met the eligibility criteria. Discrepancies between reviewers were resolved by consultation with a third author (JO) if no agreement could be reached.

The data extracted from the selected articles included the number of VSCC analyzed, the type of HPV testing, HPV prevalence, DNA sequencing technique, the gene panel used (in case of targeted NGS), and the number and frequency of identified mutations. 

## 3. Results

**Figure 1:**
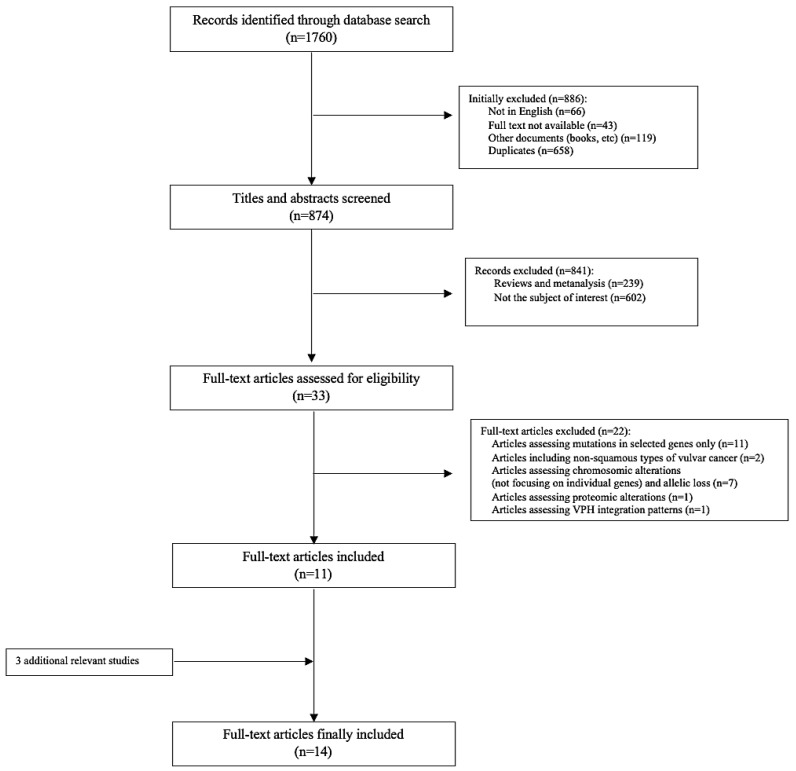
Flow diagram of publication screening and identification.

The literature review initially identified 1760 studies following the publication screening workflow described in Figure 1, and from these 886 were excluded (duplicates, book chapters, unavailable full text, and languages other than English). Among the remaining 874 titles and abstracts that were screened, 33 full-text articles were finally assessed for eligibility. Of these, 22 articles that did not meet the selection criteria were excluded, leaving 11 full-text articles, and after reviewing the references, 3 additional articles [26,27,28] were identified. 

**Figure 2:**
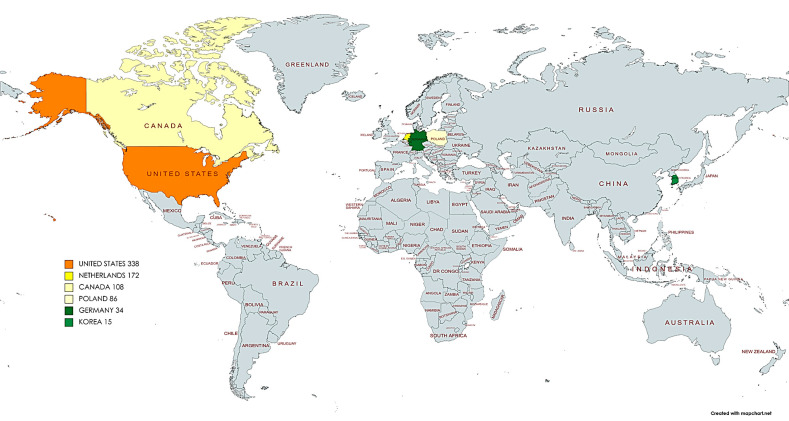
Geographical distribution and numbers of vulvar squamous cell carcinomas (VSCC) analyzed in the included studies by country.

A total of 14 studies, which explored the somatic and/or copy number mutational landscape in 749 VSCC samples from 738 patients were finally selected. The publication years ranged from 2005 to 2020. Most of the studies (n = 12; 86%) were published between 2017 to 2020 and more than one-third of them (n = 6; 43%) were released during the first COVID-19 pandemic year (2020). Seven studies (50%) were conducted in North America, six (43%) in Europe, and one (7%) in Asia. Figure 2 shows the geographical distribution of the included studies. 

Eight series (57%) were based exclusively on VSCC, whereas six (43%) included both VSCC and premalignant lesions. One study evaluated the molecular profiles in primary and metastatic VSCC in a subset of cases [29]. Eight studies (57%) analyzed only somatic mutations [25,28,30,31,32,33,34,35], two studies (14%) focused only on copy number alterations [36,37], and four (28%) included both somatic mutation profiling and analysis of copy number alterations [27,29,38,39]. Twelve studies (86%) applied NGS. Nine of the twelve (75%) NGS-based studies used targeted panels, two (17%) performed whole-exome sequencing [31,39], and one (7%) whole-genome (shallow) sequencing [37]. Both whole-exome sequencing studies included analysis of copy number alterations and one [39] additionally included the analysis of mutational signatures. Of the nine studies with targeted NGS panels, five (55%) used commercial panels, two (22%) customized panels, and two studies (22%) have not specified the panel type. Table 1 shows the main characteristics of the selected study series focused on the genomic alterations in VSCC.

The two whole-exome sequencing cohorts included VSCC matched with normal tissue, with the largest series comprised of 34 VSCC [38]. The study with the largest sample size [33] explored a total of 406 cancer-related genes in 280 VSCC samples using NGS-based hybrid capture genome profiling and analysis of mutational signatures.

HPV analysis was conducted in 13 out of the 14 studies (93%). Of the 14 studies, 9 (69%) used PCR HPV testing: unspecified PCR (3), SPF-10 (2), Amplisense HPV PCR (2), short PCR fragment L1 (1), and HPV risk assay (1). One study (8%) used an NGS-based approach, one study (8%) used HPV in situ hybridization (RNA scope), one study (8%) used only p16, and in one study (8%) HPV testing was not detailed. Of the 9 studies with HPV PCR testing, 6 studies (67%) additionally performed p16 IHC. Neither the whole-genome sequencing nor the largest NGS cohort included p16 IHC. The proportion of HPV-associated VSCC ranged between 0% [27] and 64% [30], and 9 out of the 14 studies (64%) with available HPV data compared molecular abnormalities between HPV-associated and HPV-independent VSCC. 

The prognostic implications of the molecular alterations identified in VSCC were evaluated in 7 out of 14 articles (50%). Neither the two whole-exome sequencing studies nor the largest targeted NGS-based study included follow-up data. The total number of cases analyzed, the frequencies of the alterations in each individual gene, and the number of papers in which each particular gene have been evaluated are shown in Table 2.

### 3.1. Most Frequently Analyzed and Detected Somatic Mutations in VSCC

Mutations in *TP53*, *CDKN2A*, *PIK3CA*, and *HRAS* were the most commonly analyzed and detected abnormalities. *TP53* has been assessed in 12 studies and alterations have been identified in 54% (387/712; range 33–79%) of analyzed samples. *PIK3CA* mutations have been assessed in 12 studies and the overall frequency of the mutation of this gene is 16% (112/712; range 0–34%). *HRAS* and *CDKN2A* mutations have been screened in 11 and 9 studies, respectively, and abnormalities have been identified in 9% (60/678; range 0–28%) and 26% (156/610; range 6–36%) of cases, respectively.

The most frequent (but not the most studied) somatic mutations were identified in *MUC4* (24/34; 71%), followed by *CD44* (13/24; 54%). Each of them was analyzed in a single study, in a different whole-exome-based series [36,38]. 

### 3.2. Genomic Differences Based on HPV Status 

Among the ten studies that have compared molecular abnormalities based on HPV status, two (20%) [25,39] showed that the mutational load was significantly higher in HPV-associated VSCC. However, one of the whole-exome sequencing cohorts has not identified mutational load differences [38], and two studies, including the largest NGS cohort [33], have not identified differences in terms of mutational load between the two major types of VSCC, but have shown qualitative differences in the mutational profile between HPV-associated and HPV-independent VSCC. 

The largest targeted NGS study showed that HPV-associated VSCC harbor alterations in the PI3K/mTOR pathway (*PIK3CA*, *PTEN*, *STK11*, *FBXW7*, and *SOX2*), whereas HPV-independent VSCC showed more frequent mutations in *TP53*, *TERT*, *CDKN2A*, and *CCND1*, as well as amplifications in *EGFR* and *PD-L1*. The same study estimated that at least half of the HPV-associated VSCC have a potentially targetable alteration in the PI3K/mTOR pathway [33].

Three series [28,33,38] have identified statistical differences in *TP53* alterations depending on the HPV status and four [25,29,31,39] have shown a tendency in *TP53* enrichment in HPV-independent VSCC, often combined with *CDKN2A* alterations. Two studies, both conducted by the same group [30,34], have not shown any differences for *TP53* or *CDKN2A* mutations based on HPV status.

In 2017, Watkins et al. [27] showed a significant increase of *PIK3CA* mutations in DEVIL lesions, described as an HPV-negative precursor. *PIK3CA* mutations were further confirmed not only in DEVIL but also in *TP53* wild-type dVIN [35], which also harbored *HRAS* mutations. Three years later, Tessier-Cloutier et al. focused on HPV-independent tumors, including verrucous VSCC, DEVIL, and VAAD. Strikingly, these cases were always the *TP53*-wild type but consistently harbored *PIK3CA* and *HRAS* mutations. The authors suggested a specific carcinogenic pathway different from the pathway of the typical keratinizing VSCC and dVIN [35].

### 3.3. Copy Number Variations in VSCC

One of the earliest studies conducted on VSCC focused exclusively on copy number variations in individual genes using a 122-gene panel in VSCC cell lines [36] reported a high prevalence in *TMSB10* losses (9/12, 92%) and gains in *CCND1* (8/12; 66%). The *TMSB10* copy number variations or mutations have not been confirmed in further studies, in contrast with *CCND1* alterations, which have been reported in 17% of VSCC from five studies, although with a broad range from 0% to 83%. Several studies have also shown frequent *CCND1* amplifications in HPV-independent VSCC [33,37]. Alternatively, HPV-associated VSCC harbored *TP63* and *BCL2* gains [37]. 

Whereas no differences in copy number variations loads were observed between HPV-associated and HPV-independent VSCC in the two whole-exome sequencing studies, Prieske et al. [38] identified gains in 20q and 11q as more abundant in HPV-associated and HPV-independent VSCC, respectively. Han et al. [39] showed 3q gains in HPV-associated VSCC, while the HPV-independent VSCC harbored gains in 7p and 8q and losses in 2q, and additionally identified high rates of copy number variations in *PIK3CA*. Swarts et al. [37] showed that the two types of VSCC display overlapping copy number alterations. Interestingly, this study showed that premalignant lesions and not VSCC differ significantly in terms of copy number variations. In this latter study, gains in chromosome 1 were identified as a risk factor for progression from vulvar high-grade squamous intraepithelial lesions to VSCC. 

### 3.4. Prognostic Role of Molecular Alterations in VSCC

One of the earliest studies [31] showed that mutations in both *TP53* and *HRAS*, or *CDKN2A*, related to HPV-independent VSCC, were associated with a significantly worse prognosis. Zieba et al. [30] reported that neither HPV status nor mutations were associated with VSCC patient progression. Nooij et al. [25] showed a higher local recurrence rate of patients with HPV-independent *TP53*-mutated VSCC, compared with HPV-independent *TP53*-wild type VSCC and HPV-associated VSCC. Tessier-Cloutier et al. [35] reported worse overall survival in cases with *TP53* and *PIK3CA* co-mutations. The remaining three study series have not shown solid evidence of the prognostic impact of the explored gene mutations. 

### 3.5. Potential Molecular Therapeutic Targets to Treat VSCC

In most of the included studies, the authors suggested therapeutic molecular targets based on the molecular alterations identified. Targeting the PI3K/AKT/mTOR pathway was the most frequently proposed strategy among the reviewed series [27,28,29,30,31,33,34,35]. Williams et al. [33] suggested that patients with *KMT2D* mutations might benefit from aurora kinase inhibitors. A few authors [28,31] proposed that the use of combined regimens (i.e., MEK inhibitors and PI3K inhibitors, mTOR and MEK inhibitors) might be useful to treat VSCC, instead of only targeting the PI3K pathways.

Other identified potential therapeutic targets involved *NOTCH-1* [25], *FGFR* [28], *MET*, and *BRAF* [35]. Kunjoonju et al. suggested that *TMSB10*, *CTNNB1*, *BCL2*, *CCND1*, and *IL12A* might be key molecular targets in VSSC [36], while Watkins et al. [27] highlighted that *EGFR-* mutated patients might benefit from targeted therapy. 

## 4. Discussion

A growing number of research studies have focused on the genomic landscape in VSCC, particularly in the last four years. The analysis of these studies shows a marked variation in the number of mutations, the specific mutated genes, and the frequencies of these mutations. Unfortunately, there were notorious methodological differences between the studies, and consequently, their results might not be comparable, which represents the main limitation of the present study. Notably, the number of cases and the set of genes analyzed was limited in almost all series. Indeed, the two whole-exome sequencing series included no more than 50 samples in total, whereas the largest targeted NGS study explored 406 genes, which constitutes less than 2% of the genome coverage of any of the whole-exome sequencing studies, which might prevent obtaining solid molecular profiles.

While some series have suggested that HPV-independent tumors have a larger mutational load [25,39], other series [33,38] have indicated that the mutational load does not significantly differ by HPV status. However, the variation in the molecular techniques and strategies to detect HPV and, therefore, the comparisons between the different studies, might be biased. More importantly, p16 staining, a well-characterized surrogate marker of HPV status in VSCC [11], has been used only in half of the studies, while the combination of p16 and HPV PCR, probably the best strategy to conclusively assign a case as HPV-associated or HPV–independent [9], has been used in less than half of the series. It is particularly notorious there was a lack of the use of p16 staining in the whole-exome sequencing studies as well as in the largest NGS cohort. Thus, the analysis based on HPV status might be limited. The few studies that exclusively used PCR-based HPV testing [30,34] reported no clear genomic differences between HPV-associated and HPV-independent VSCC, but the authors acknowledge that the HPV tests used in the studies were not designed to be used in formalin-fixed, paraffin-embedded tissue [30]. Therefore, the hypothesis of similar oncogenic mechanisms for HPV-associated and HPV-independent VSCC lacks a solid basis.

Despite these limitations, the genomic landscape of VSCC is expanding beyond the well-known mutations in tumor suppressors *TP53* and *CDKN2A*, biomarkers that are difficult to target [40]. Mutations in the PI3K/AKT/mTOR pathway, apart from *TP53*, including *HRAS*, *KRAS*, *PIK3CA*, *KMT2D*, *PTEN*, and *FBXW7*, have been consistently reported across different study series. One of the whole-exome sequencing studies [39] showed that somatic mutations of *PIK3CA*, combined with the copy number variations in the same gene, comprised more than half (60%) of all molecular alterations, irrespective of the HPV status. Indeed, one of the systematic reviews [41] highlighted the PI3K pathway as the most important genomic abnormality in VSCC. Notably, most of the mutations involved the PI3K/AKT/mTOR pathway were more frequently found in HPV-associated VSCC in the largest study [33]. However, this study was based on the PCR-only strategy for HPV identification, with no p16 IHC. Thus, this correlation with HPV status has to be interpreted cautiously.

Several of the genes of the PI3K/AKT/mTOR pathway, including *PIK3CA*, *PTEN*, and *FBXW7*, have been described in the Drug Gene Interaction database as targetable by known drugs. Accordingly, several authors proposed targeting the PI3K/AKT/mTOR pathway. For instance, patients with *KMT2D* mutations might benefit from aurora kinase inhibitors, as suggested by Williams et al. [33], and recently shown in head, neck, and cervical cancer [42]. Nevertheless, the prognostic or therapeutic roles of the abnormalities in this pathway in VSCC are yet to be elucidated [43]. 

Besides the genes directly involved in the PI3K/AKT/mTOR pathway, other genes, such as *NBPF1* and *TSC2*, can have activating or inhibiting interactions with this cascade. Although identified in a single study series in this review, *NBPF1* has tumor growth inhibitory effects through the inhibition of the PI3K signaling pathway [44]. *TSC2* losses also lead to the enhancement of mTOR activity [45]. Therefore, the role of the PI3K/AKT/mTOR cascade likely plays a bigger role than originally thought.

Interestingly, the largest NGS cohort [33] identified significant rates of *NOTCH-1* mutations in HPV-independent tumors (19%). The Notch signaling pathway is known as one of the key players in maintaining normal tissue homeostasis [46], but similarly to the PIK3CA/AKT/mTOR pathway, its prognostic and therapeutic implications are far from clear in solid cancers. Interestingly, high rates (71%) of *NOTCH-1* mutations have been shown in a whole-exome sequencing study of squamous cell carcinomas of the penis [47], a male tumor with many similarities with VSCC (dual HPV-associated/HPV-independent pathway, similar precursor lesions). Moreover, the authors identified mutations in the PI3K pathway in one-third of the tumors. These shared findings between VSCC and penile cancer might open possibilities for the enrollment in trials exploring the role of *NOTCH-1* mutations as predictors of response to PI3K/mTOR inhibitors [48]. 

The evidence of *EGFR* amplifications in 11% of HPV-independent VSCC, as shown by Williams et al. [33], might open doors to prognostic stratification or treatment with Cetuximab [49,50]. A phase II clinical trial assessing the role of erlotinib (anti-*EGFR* tyrosine kinase inhibitor) in VSSC has shown an acceptable toxicity with a significant clinical response (27.5% of patients showed partial response and 40% stable disease), but with limited sustained response rates [51]. Alternatively, as *CCND1* amplifications are also most frequently seen in HPV-independent VSCC [33,36], it might be worth exploring the potential additive oncogenic effects of *EGFR* and *CCND1* alterations, as recently shown in oral squamous cell carcinomas [52].

Another intriguing observation, recognized in both the whole-exome sequencing as well as the largest NGS cohorts, are abnormalities in *FBXW7*, a p53-dependent tumor suppressor gene frequently mutated in other female genital tumors, such as endometrial and cervical cancers [53]. *FBXW7* is a modulator of NOTCH signaling cascade and recent studies have implicated *FBXW7* status in chemoresistance [54]. However, while the largest NGS study [33], and one of the whole-exome sequencing series, identified *FBXW7* mutations predominantly in HPV-associated tumors, in the second whole-exome sequencing study [38] these mutations were restricted to HPV-independent VSCC. 

Mutations in mucins, including *MUC16* (formerly known as *CA125*), have been frequently detected in a whole-exome sequencing-based study [38]. Nevertheless, these mutations should be confirmed in new studies using whole-exome sequencing or modern NGS-based tools. Stimulatingly, *MUC16* has been shown to be altered only in gynecological malignancies and other benign conditions [55] and has been recognized as a tumor biomarker and a novel target for cancer therapy [56].

Curiously, none of the series confirmed copy number variations in *TMSB10* and *IL12A*, identified with high frequencies by Kunjoonju et al. [36]. However, this study used a small subset of 12 VSCC cell lines and not formalin-fixed, paraffin-embedded, or frozen tissue as most of the other series did. Similarly, a subset of somatic mutations, including *MUC4* or *CD44*, has been reported only by one of the whole-exome studies. Thus, the methodology employed by these studies might not be robust.

The prognostic role of genomic alterations is limited to the few most recurrent genes. It is of note that the prognostic differences were identified for combinations of mutations rather than for individual alterations. The co-mutations with worse prognoses mostly consisted of *TP53* combined with *HRAS*, *CDKN2A*, or *PIK3CA* mutations [31,35]. As *TP53* is more commonly mutated in HPV-independent VSCC [38], these findings are in line with the increasing evidence on the worse prognoses of HPV-independent VSCC [57].

In 2017, Nooij et al. [25] suggested that a subset of HPV-independent VSCC (HPV-negative, *TP53*-wild type) should be considered as a distinct etiopathogenic, morphologic, and molecular subtype, characterized by *NOTCH-1* and *HRAS* mutations. More recently, Tessier-Cloutier et al. have provided evidence indicating that a particular variant of VSCC, verrucous VSCC, and two precursor lesions, DEVIL and VAAD, might be part of the spectrum of this distinct HPV-negative *TP53*-wild type pathway and that all of these lesions harbor *HRAS* and *PIK3CA* mutations [35]. However, neither the whole-exome sequencing cohorts nor the largest NGS series specifically analyzed these lesions, and the evidence indicating that they truly represent a specific entity is still limited. Moreover, the clinical behavior of these lesions is still poorly understood [1,16]. 

In conclusion, although with the recent effort in characterizing the genomic landscape of VSCC, much still remains unknown on the molecular mechanisms involved in the pathogenesis of this tumor. Comparisons between existing series on VSCC are limited by different sample sizes, heterogeneous HPV detection, and tumor DNA sequencing methods. Despite it being known that a number of mutations are druggable, the clinical utility of them is still unknown in patients with VSCC. Large-scale, ideally multicentric studies, with a solid HPV testing strategy p16 and p53 IHC, a strong follow-up component to further analyze possible prognostic implications related to genomic mutations, as well as clinical trials analyzing the possibility of gene-targeted therapies, are needed to elucidate the specific roles of known and newly described mutations, or combinations of them.

## Figures and Tables

**Table 1:** Main characteristics of the studies analyzing the genomic alterations in vulvar squamous cell carcinomas (VSCC).

	Source	Research Paper	Year	Country	Type of Sample	Type of Genomic Analysis	Gene Panels	Number of Targeted Genes	N (VSCC Samples)	HPV Prevalence	Most Frequent Individual Gene Alteration	Differences in Overall Mutational Frequency by HPV Status	Genes More Altered in HPV+ VSCC	Genes More Altered in HPV-VSCC
1	[36]	Kunjoonju et al.	2005	USA	Cell lines	MLPA	Customized	122	13	ND	*TMSB10* (92%), *CCND1* (83%), *IL12A (67%), CTNNB1* (67%), *BCL2* (58%)	-	N/A	N/A
2	[31]	Trietsh et al.	2014	The Netherlands	FFPE blocks	Sanger Mass spectometry	GynCarta 2.0 panel	13	107	16.2%	*TP53 (54%), CDKN2A (13%), HRAS (9%), PIK3CA (7%), PPPR1A (3%), KRAS (1%), PTEN (1%)*	*-* (not analyzed)	*-*	*TP53, CDKN2A, HRAS, PIK3CA, KRAS*
3	[25]	Nooij et al.	2017	The Netherlands	FFPE blocks	Targeted NGS	Customized	17	36	22.2%	*TP53 (58%), NOTCH1 (33%), HRAS (29%), KMT2D (11%)*	Yes (higher in HPV-VSCC)	*-*	*TP53, NOTCH-1, HRAS*
4	[28]	Weberpals et al.	2017	Canada	FFPE blocks	Targeted NGS	Ion AmpliSeq Cancer Hotspot v2 Panel	50	43	51.2%	*TP53 (35%), PIK3CA (23%), HRAS (14%), KIT, CDKN2A (12%), FGFR3 (9%)*	No	*FGFR3*	*TP53,* *PIK3CA,* *HRAS,* *CDKN2A*
5	[27]	Watkins et al.	2017	USA	FFPE blocks	Targeted NGS	N/S	300	14	0.0%	*TP53 (79%), CDKN2A (36%), PIK3CA (14%), KMT2D (14%), CCND1 (14%), EGFR (7%)*	N/A	N/A	N/A
6	[39]	Han et al.	2018	Korea	FFPE blocks (n = 14 cases) Frozen tissue (n = 1)	NGS	N/A	Whole exome	15	40.0%	*TP53 (33%), FAT1 (27%), APC (20%), CASP8 (20%), PIK3CA (13%), FBXW7 (13%), NOTCH-1 (13%), BRCA2 (13%), EP300 (13%)*	Yes (higher in HPV- VSCC)	*PIK3CA, FBXW7,*	*TP53, CDKN2A, HRAS, FAT1, APC*
7	[37]	Swarts et al.	2018	The Netherlands	FFPE blocks	NGS	N/A	Whole genome shallow sequencing	24	45.8%	*TP63 (46%), JAG1 (33%), CD44 (54%), MET (77%), DSC/DSG (38%), RBFOX1 (38%)*	No	*TP63* gains	*CCND1* amplifications
8	[30]	Zieba et al.	2018	Poland	Frozen samples Celll lines	Targeted NGS	Ion AmpliSeq Cancer Hotspot v2 Panel	50	81	64.0%	*TP53 (44%), CDKN2A (23%), PIK3CA (9%), FBXW7 (6%), HRAS (6%),*	No	*AKT1,* *FGFR3,* *SMAD4,* *JAK3*	*FLT3,* *GNAQ*
9	[34]	Zieba et al.	2020	Poland/ The Netherlands	FFPE blocks	Targeted NGS	Ion AmpliSeq Cancer Hotspot v2 Panel	50	10	40.0%	*TP53 (70%), CDKN2A (30%)*	No	*-*	*-*
10	[35]	Tessier-Cloutier et al.	2020	Canada	FFPE blocks	Targeted NGS	N/S	33	33	6.1%	*TP53 (72%), PIK3CA (34%), HRAS (28%), PTEN (6%), MET (9%), BRAF (3%)*	No	*-*	*-*
11	[38]	Prieske et al.	2020	Germany	Frozen tissue	NGS	N/A	Whole exome	34	35.3%	*TP53 (56%), MUC4 (71%), TTN (29%), ZNF717 (29%), PIK3CA (11%), KMT2D (11%), SYNE2 (15%), SYNE1 (13%), FBXW7 (9%), NSD1, NBPF1 (21%), CDKN2A (6%)*	Only for CNA	20q gains	*TP53* 11q gains
12	[33]	Williams et al.	2020	USA	Blood FFPE blocks	Targeted NGS (CGP)	Foundation One platform	406	280	36.3%	*TP53 (55%), CDKN2A (36%), TERTp (49%), EGFR (9%), PIK3CA (22%), CCND1 (15%), NOTCH-1 (14%), CDKN2A (36%), PTEN (6%), FBXW7 (6%)*	No	*PIK3CA,* *PTEN, EP300, STK11,* *AR,* *FBXW7,* *KMT2D, BAP1*	*TP53,* *TERTp,* *CDKN2A,* *CCND1,* *FAT1, NOTCH1,* *EGFR* *PDL-1/PDL-2*
13	[32]	Pors et al.	2021	Canada	FFPE blocks	Targeted NGS	Customized	33	33	0.0%	*TP53 (645), HRAS (6%), PIK3CA (6%), PTEN (3%), GNAS (3%) EGFR (3%)*	N/A	N/A	N/A
14	[29]	Xing et al.	2020	USA	FFPE blocks	Targeted NGS (CGP)	Ion ampliseq Oncomine Comprehensive v.2	143	42 *	37.5%	*TP53 (62%), CDKN2A (27%), PIK3CA (15%), HRAS (8%), NOTCH-1 (8%)*	(not analyzed)	*PIK3CA*	*TP53,* *CDKN2A,* *HRAS,* *NOTCH-1,* *BIRC3* amplifications

CNA; copy number alterations; FFPE: formalin-fixed paraffin-embedded; HPV: human papillomavirus; NGS: Next-generation sequencing. WES: whole-exome sequencing; CGP: comprehensive genomic profiling; N/A: not applicable; ND: not determined; N/S: not specified; MLPA: multiplex ligation-dependent probe amplification assay; USA: United States of America; * only data from 26 VSCC was available to be included this review.

**Table 2:** Frequencies of identified alterations in individual genes stratified by the number of articles that performed molecular analyses in vulvar squamous cell carcinomas (VSCC).

Gene	Number of VSCC with the Gene Alteration	Number of VSCC Assessed	Overall Frequency	Frequency Range	Number of Articles
*TP53*	387	712	54.4%	33–79%	12
*PIK3CA*	112	712	15.7%	0–34%	12
*HRAS*	60	678	8.8%	0–28%	11
*CDKN2A*	156	610	25.6%	6–36%	9
*PTEN*	26	647	4.0%	0–6%	9
*CTNNB1*	12	326	3.7%	0–67%	8
*EGFR*	33	456	7.2%	0–29%	7
*KRAS*	10	589	1.7%	0–23%	7
*NOTCH1*	57	482	11.8%	0–33%	7
*FBXW7*	27	434	6.2%	0–13%	6
*FGFR3*	11	474	2.3%	0–9%	6
*RB1*	13	446	2.9%	0–7%	6
*STK11*	19	452	4.2%	0–7%	6
*ATM*	10	164	6.1%	0-67%	5
*BRAF*	1	267	0.4%	0–3.3%	5
*CCND1*	63	365	17.3%	0–83%	5
*ERBB2*	7	437	1.6%	0–3%	5
*ERBB4*	8	173	4.6%	0–50%	5
*MET*	14	191	7.3%	0–9%	5
*RET*	2	171	1.2%	0-7%	5
*B2M*	6	26	23.1%	0–50%	2
*BCL2*	14	36	38.9%	29–58%	2
*PRKDC*	7	27	25.9%	0–58%	2
*BIRC2*	6	13	46.1%	NA	1
*CASP1*	5	13	38.5%	NA	1
*CASP6*	6	13	46.1%	NA	1
*CD44*	13	24	54.2%	NA	1
*CREBBP*	3	15	20.0%	NA	1
*DSC*	9	24	37.5%	NA	1
*EMS1*	4	13	30.8%	NA	1
*HIF1A*	7	24	29.2%	NA	1
*IL6*	6	13	46.1%	NA	1
*ILI2A*	7	13	61.5%	NA	1
*JAG1*	8	24	33.3%	NA	1
*MUC4*	24	34	70.6%	NA	1
*NBPF1*	7	34	20.6%	NA	1
*NCOA3*	5	13	38.5%	NA	1
*NKFB1*	5	13	38.5%	NA	1
*NRG1*	3	15	20.0%	NA	1
*PRFKDC*	6	13	46.1%	NA	1
*PTPRD*	5	24	20.8%	NA	1
*RBFOX1*	8	24	33.3%	NA	1
*RBFOX3*	7	24	29.2%	NA	1
*TERTp*	136	280	48.6%	NA	1
*THBS1*	5	13	38.5%	NA	1
*TMSB10*	11	13	84.6%	NA	1
*TTN*	10	34	29.4%	NA	1
*ZFHX3*	3	15	20.0%	NA	1
*ZNF717*	10	34	29.4%	NA	1

N/A: not applicable.

## Data Availability

All the data is shown in the manuscript.
